# The Association between Aids Related Stigma and Major Depressive Disorder among HIV-Positive Individuals in Uganda

**DOI:** 10.1371/journal.pone.0048671

**Published:** 2012-11-27

**Authors:** Dickens Akena, Seggane Musisi, John Joska, Dan J. Stein

**Affiliations:** 1 Department of Psychiatry and Mental Health, University of Cape Town, Cape Town, South Africa; 2 Department of Psychiatry, Makerere University, Kampala, Uganda; University of Ottawa, Canada

## Abstract

**Background:**

Major depressive disorder in people living with HIV/AIDS (PLWHA) is common and may be associated with a number of factors, including AIDS-related stigma, decreased CD4 levels, increased opportunistic infections and sociodemographic variables. The extent to which AIDS-related stigma is associated with major depressive disorder among PLWHA has not been well studied in sub-Saharan Africa. The objective of this study was to examine the associations between major depressive disorder, AIDS-related stigma, immune status, and sociodemographic variables with the aim of making recommendations that can guide clinicians.

**Methods:**

We assessed 368 PLWHA for major depressive disorder, as well as for potentially associated factors, including AIDS-related stigma, CD4 levels, presence of opportunistic infections, and sociodemographic variables.

**Results:**

The prevalence of major depressive disorder was 17.4%, while 7.9% of the participants had AIDS related stigma. At multivariable analysis, major depressive disorder was significantly associated with AIDS-related stigma [OR = 1.65, CI (1.20–2.26)], a CD4 count of ≥200 [OR 0.52 CI (0.27–0.99)], and being of younger age [0.95, CI (0.92–0.98).

**Conclusions:**

Due to the high burden of major depressive disorder, and its association with AIDS related stigma, routine screening of PLWHA for both conditions is recommended. However, more research is required to understand this association.

## Introduction

HIV/AIDS is one of the biggest health crises the world faces today, with close to two-thirds of all people living with HIV/AIDS (PLWHA) residing in sub-Saharan Africa [1]. The prevalence of HIV/AIDS in Uganda in 2012 stood at 7.2%, with women disproportionately represented [2]. The recent ministry of health demographic survey put the prevalence of HIV/AIDS at 8.3% in women and 6.1% in men [2]. Treatment coverage for PLWHA is poor in Uganda, with only half of PLWHA accessing antiretroviral therapy (ART) [3].

Among PLWHA, depressive disorder is the commonest neuropsychiatric disorder, occurring at rates two to three times higher than in HIV-negative patients [4]–[6]. The prevalence of depressive disorder among PLWHA in Uganda has been reported as 20–40% [7]–[10]. Depressive disorder in PLWHA has been associated with several critical adverse health- related outcomes. Previous work has documented poor adherence to medications, including ART in depressed PLWHA [11]–[13]. Other researchers have also documented that PLWHA suffering from depression progress faster from HIV to AIDS compared to non-depressed PLWHA [14]–[17]. Compared to depressed HIV negative persons, PLWHA who suffer from depression have also been shown to have a poor quality of life [18].

The development of depressive disorder among PLWHA is likely the result of a combination of biological and sociodemographic variables [14], [19]–[22]. Some of these biological variables may be relatively unique to PLWHA, such as AIDS-related stigma [20], [21], compromised immune status (low CD4 counts) and increased opportunistic infections [19], [23], [24]. However, sociodemographic variables including gender, low education and lack of employment have been associated with depression in both HIV negative and positive populations [22], [25], [26]

AIDS- related stigma is prominent among PLWHA and has been shown to negatively impact on the quality of life, leading to poor functioning of affected individuals [27], [28]. Research has also shown that PLWHA who have stigma are less likely to access HIV care services [27], [29], [30],and could have poor psychological wellbeing [21], [31]. Some work done in South Africa has documented increased burden of AIDS stigma and its association with mental disorders [20], [32]. Despite its prominence, AIDS related stigma among PLWHA is often unidentified [28], .

The dual existence of AIDS related stigma and major depressive disorder among PLWHA could lead to a number of adverse health outcomes. However, little work has been done to particularly assess the association between AIDS related stigma and major depressive disorder in sub-Saharan Africa [20], [32], [33]. Examining the relationship between AIDS related stigma and major depressive could prove useful in raising clinician's awareness about the need to holistically assess PLWHA who present at PHC.

Similarly, literature about the other factors that may be associated with major depressive disorder, including immunological and sociodemographic variables in PLWHA is less consistent. Thus while some work has reported that a low CD4 count is associated with having depression in PLWHA [15], [17], other researchers have found otherwise [22], [34]. Research findings about the association between major depressive disorder and female gender [22], [35], being of younger age [35]–[37] and unemployment [37] have equally shown inconsistencies. Examining the association between depression and these factors is important since some of them have been shown to influence health outcomes in PLWHA [15], [19], [22].

In this study, we investigated the extent to which major depressive disorder was associated with AIDS-related stigma, and a number of other variables in PLWHA with the aim of making recommendations that can guide clinicians.

## Methods

### Study design and setting

This was a cross sectional study which took place at the Nsambya Hospital Home care department, an HIV-PHC facility 3 km from Kampala city, between the months of April and June 2011.

### Study population

The study population consisted of PLWHA who were medically stable and had been in care for atleast 6 months. Patients were excluded if they presented with a mental illness requiring admission.

### Study procedure

About 150–200 patients attend the clinic daily; each of them is given a number based on time of arrival (1–200 for example). Using EPIDATA, we randomly generated 15–20 numbers daily, each number belonging to a potential clinic attendee, who would be approached and informed consent obtained. Triage nurses then administered the patient health questionnaire-9(PHQ-9) [38] to screen for depression.

Research assistants, who were medical Doctors and holders of an MBChB degree, and were trained by the principal investigator, administered the study instruments. The presence of a current major depressive disorder, according to the Mini International Neuropsychiatric Inventory (MINI) [39] depression module was confirmed by the research assistants. The presence of bipolar depression was ruled out by asking whether patients had ever had an episode of mania or hypomania. Such patients (5 in number) were not included in the final analysis. The research assistants also administered the sociodemographic questionnaire and the AIDS-related stigma scale to all consented patients. The PHQ-9 was administered as part of a validation study, the findings of which will be reported elsewhere. Research assistants abstracted the most recent CD4 count level from patient's charts. All the questionnaires were translated from English into the local language (Luganda), and administered in either language, depending on which of the two was understood by the patient.

### Ethical approval

The study was approved by the Makerere University School of Medicine Ethics committee and the University of Cape Town Health Sciences Human Research Ethics Committees. Study participants received a transport refund of 10,000 Ugandan shillings (approximately4$).

### Study instruments

The MINI was designed as a brief structured interview for diagnosing the major Axis I psychiatric disorders in DSM-IV and can be administered in 18.7±11.6 minutes (median 15 minutes). The MINI has been used in a number of studies as a diagnostic instrument among PLWHA in Uganda [7], [35], [36], [40].

AIDS related stigma scale [41] is a 9 item that developed for use in sub-Saharan Africa. It was validated among 2300 patients, and showed good psychometric properties. The internal consistency of the scale was 0.75, and was time stable over three months r = 0.67. The 9- item AIDS related stigma scale taps into a broad range of stigmatizing beliefs including negative beliefs towards self and others (internalized and enacted variables of stigma) [41].

Sociodemographic information, presence of opportunistic infections and CD4 counts was collected from all participants using a standardized questionnaire.

The PHQ-9 was adapted from the primary care evaluation of mental disorders (PRIME MD) screening questionnaire for depressive symptoms, and has 9 questions with a score ranging from 0 to 3 for each question (maximum score of 27). A threshold score of 10 or higher is considered to indicate mild major depressive disorder, 15 or higher indicates moderate major depressive disorder, and 20 or higher severe major depressive disorder. The internal consistency of the PHQ-9 was 0.65 [42] The PHQ-9 has not been validated in Uganda; however, it was validated among PLWHA in Kenya providing good psychometric properties with a coefficient alpha of 0.78. [43].

### Study measures

A diagnosis of a major depressive disorder was arrived at if participants had atleast 5 of the 9 DSM-IV-TR symptoms for major depression, and were judged to have social and occupational impairments as a result of the symptoms. Persons diagnosed as depressed were referred to the attending clinic medical officer for treatment. AIDS related stigma was diagnosed if patients positively endorsed atleast 5 out of the nine questions. Persons diagnosed with AIDS-related stigma were referred to the hospital counsellor.

### Data Analysis

Data was analysed using STATA 11.2 [44]. We initially compared individuals with and without major depressive disorder using bivariate analysis; variables that were statistically significant at bivariate analysis were then entered into a multivariable model following a stepwise hierarchical format. Logistic regression was used to test for significant associations, controlling for age and gender. Major depressive disorder was the dependent variable, with AIDS related stigma, immune status and sociodemographic variables as independent variables. We also assessed whether the sociodemographic and clinical variables were confounding the association between AIDS related stigma and Major depression.

## Results

The mean age of the respondents was 38.8 years (SD 9.81; range 18–71). Of the 368 participants, 72% were female. About two thirds (65.5%) had some form of employment, and only 196 (53.8%) had attained at least secondary education. Mean CD4+ was 329.70, (SD 183.95; range 1–999). Median CD4+ was 336, inter-quartile range 25–75% (250–356). The prevalence of major depressive disorder was 17.4%. Twenty nine out of the 368 participants (7.9%) had AIDS related stigma. (See table 1).

**Table 1:** Sociodemographic and clinical characteristics of the respondents.

Variable	Frequency(%)
**Major depression**	
Yes	64(17.39%)
No	304(82.61%)
**Gender**	
Male	107(29%)
Female	261(71%)
**Age**	
18–29	65(17.7%)
30–39	144(39.1%)
40–49	108(29.3%)
50+	51(13.8%)
Age(Mean(sd))	38.84(10.08)
**AIDS stigma**	
Yes	29(7.88%)
No	339(92.1%)
**Marital status**	
Not married	229(62.2%)
Married	139(37.8%)
**Employment**	
Employed	241(65.5%)
Not employed	127(34.5%)
**Education status**	
Primary education	170(46.2%)
Secondary Education	198(53.8%)
**CD4 counts**	
<200	61(16.6%)
>201	307(83.4%)
**CD4(**Mean(sd))	328.22(182.36)
Median	336 IQR 25–75%(250–356)
**Opportunistic Infection**	
Yes	154(41.8%)
No	214(58.2%)
**WHO stage**	
I	92(25%)
II	135(36.7%)
III	95(25.8%)
IV	41(11.1%)

At bivariate analysis, participants with major depressive disorder were more likely to have AIDS-related stigma [OR 1.61, CI (1.19–2.17)], an opportunistic infection [OR 1.89, CI (1.05–3.38)], and a lower CD4 ≥200) [OR 0.52 CI (0.27–0.99)]. Being of younger age [OR 0.42 CI (0.19–0.92)], lacking employment [OR 0.53, CI (0.30–0.91)] and having a lower education [OR 0.48, CI (0.27–0.84)] were also associated with depression at bivariate analysis. (See table 2).

**Table 2:** Bivariate analysis of the sociodemographic and clinical characteristics of the respondents.

Variable	Depressed	Not depressed	P-value
	(frequency, %)	(frequency, %)	
**Gender**			
Male	18 (16.82)	89(83.18)	
Female	46(17.62)	215(82.38)	0.854
**Age**			
18–29	17(26.15)	48 (73.85)	
30–39	27(18.75)	117(81.25)	0.068
40–49	14(12.96)	94(87.04)	**0.014** *
50+	6(11.76)	45(88.24)	**0.034** *
**AIDS stigma**			
Yes	10 (34.48)	19(65.52)	**0.002** *
No	54(15.93)	285(84.07)	
**Marital status**			
Not married	37 (16.16)	192(83.84)	
Married	27(19.42)	112(80.58)	0.423
**Employment**			
Employed	34(14.11)	207(85.89)	
Not employed	30(23.62)	97(76.38)	**0.023** *
**Education level**			
Primary	39(22.94)	131(77.06)	
Secondary	25(12.63)	173(87.37)	**0.010** *
**CD4 counts**			
≤200	16 (26.23)	45(73.77)	
≥201	48(15.64)	259(84.36)	**0.049** *
**Opportunistic**			
**Infection**			
Yes	19(12.34)	135(87.66)	**0.032** *
No	45(21.03)	169(78.97)	
**WHO stage**			
I	13(14.13)	79 (85.87)	
II	26(19.26)	109(80.74)	0.316
III	15(15.79)	80(84.21)	0.751
IV	10(24.39)	31(75.61)	0.153

* denotes p value <0.05 and statistical significance.

We included all statistically significant variables in the logistic regression model, and used a stepwise hierarchical model analysis method. In the final model, major depressive disorder was associated with AIDS-related stigma [(OR = 1.65, CI 1.20–2.26)], CD4+ counts of ≥200 [OR 0.43, (CI 0.20–0.91)] and a younger age [OR0.95, CI 0.92–0.98)]. (See table 3).

**Table 3:** Multivariable analysis showing associations between major depression, AIDS related stigma and CD4 counts.

Variable	Depressed	Unadjusted	95% CI	Adjusted	95% CI
	(frequency, %)	OR		OR	
**Age**					
18–29	17(26.15)				
30–39	27(18.75)	0.65	0.32–1.30		
40–49	14(12.96)	**0.42** *	**0.19–0.92**	**0.95** *	**0.92–0.98**
50+	6(11.76)	0.37	0.13–1.03		
**AIDS stigma**					
Yes	10 (34.48)	**1.61** *	**1.19–2.17**	**1.65** *	**1.20–2.26**
No	54(15.93)				
**CD4 counts**					
≤200	16 (26.23)				
≥201	48(15.64)	**0.52** *	**0.27–0.99**	**0.43** *	**0.20–0.91**

* denotes p value <0.05 and statistical significance. Odds ratios adjusted for sex and age.

## Discussion

Major depressive disorder was prevalent in our study population, occurring in 17.4% of the participants. Previous studies conducted among PLWHA in Uganda have reported higher depression prevalence [9], [45]; findings which could be explained by the fact that in those studies, the diagnosis of depression was made using a screening instrument, rather than a diagnostic one. Our population comprised of medically stable participants who were generally healthier, and this could explain the lower prevalence compared to the other Ugandan studies..

AIDS-related stigma, a condition that has been associated with adverse health outcomes in PLWHA was equally prevalent in the study population. Our finding about AIDS related stigma is also in keeping with previous studies that have reported a high burden of stigma in PLWHA [46]–[48].

We found an association between major depressive disorder and AIDS related stigma, meaning that both conditions may be present in the same HIV-positive individual attending PHC. Our findings are in keeping with a previous study that documented an association between depression and stigma in PLWHA [17]. Poor psychosocial functioning, the presence of opportunistic infections, poor immune status and the fear of dying from a chronic illness could explain the existence of either of these conditions, as well as their association with each other. Previous studies have reported that stigma among PLWHA is associated with poor psychosocial functioning [21], [49]. It's possible that people with poor psychological functioning may develop depression.

Similarly, the presence of opportunistic infections and poor immune status has been associated with depression in PLWHA [17], [19], [45]. It can also be argued that depressed PLWHA who have opportunistic infections and low CD4+ counts could develop stigma as a result of their condition. The fear of dying from a chronic illness may also explain the presence of both conditions. However, the cross-sectional nature of our study makes it difficult to establish causality, and the direction of the development of each condition.

Our findings regarding the association between major depressive disorder and low CD4 counts are in keeping with previous studies [17], [45], [50]. These findings could be explained by the fact that late stage disease (manifested by low CD4 counts) may have an aetiological role in the development of depression among PLWHA. The presence of depression in PLWHA could also lead to a decline in CD4 levels; such an association has been previously documented [17], [50]. It's also possible that the sicker PLWHA become, the more likely they are to report symptoms of major depressive disorder. More work is needed to examine such hypotheses.

The association between major depressive disorder and younger age contradicts previous studies where major depressive disorder was particularly common in older people attending PHC services [35], [36], [51]. Perhaps the different contexts in which HIV/AIDS manifests could explain such differences. Specific neurobiological factors may play a role in contributing to major depressive disorder in older subjects; further work is needed to explore this hypothesis.

A number of limitations in this study deserve emphasis. We utilised a cross-sectional design, so that causality cannot be fully addressed. A longitudinal follow-up study could provide better insight into the precise nature of the relationship between depression, and the studied factors. That said, PLWHA should be assessed for both major depressive disorder and AIDS related stigma since both conditions may present concurrently in the same individual.

Secondly, the study was conducted in a single PHC site, and may not be representative of the burden of major depressive disorder in PLWHA in Uganda.

Thirdly, we didn't abstract information regarding patients being on ART, despite the fact that a number of PLWHA at the study site were accessing ART. This information could have given us better insight into its relationship with depression and stigma.

Fourth, the instruments we used including the MINI, AIDS stigma scale, and the PHQ-9 haven't been validated in Uganda. This could have led to some inaccuracies in our findings. However, a number of studies have been conducted in Uganda using the MINI, and have reported similar prevalence findings to our study [5], [9], [52], [53]

Despite these limitations, this study reports on the association between major depression, AIDS stigma and a number of variables among PLWHA in sub-Saharan Africa. Clinicians working in HIV settings should regularly assess for both depression and stigma among clinic attendees, since these conditions may be present concurrently in PLWHA.

In conclusion, due to the high burden of major depressive disorder, and its association with AIDS related stigma among PLWHA, routine screening of PLWHA for both conditions is recommended. However, further work may be required to understand the complex relationships between AIDS stigma and major depressive disorder. Further work to disentangle the relationships between major depressive disorder and low CD4 counts is equally needed.
